# Supplementary figures and images for: Long-term responses of Icelandic Arctic foxes to changes in marine and terrestrial ecosystems
Source: PLoS One. 2023 Oct 4;18(10):e0282128. doi: 10.1371/journal.pone.0282128 (PMC10550166; doi:10.1371/journal.pone.0282128)

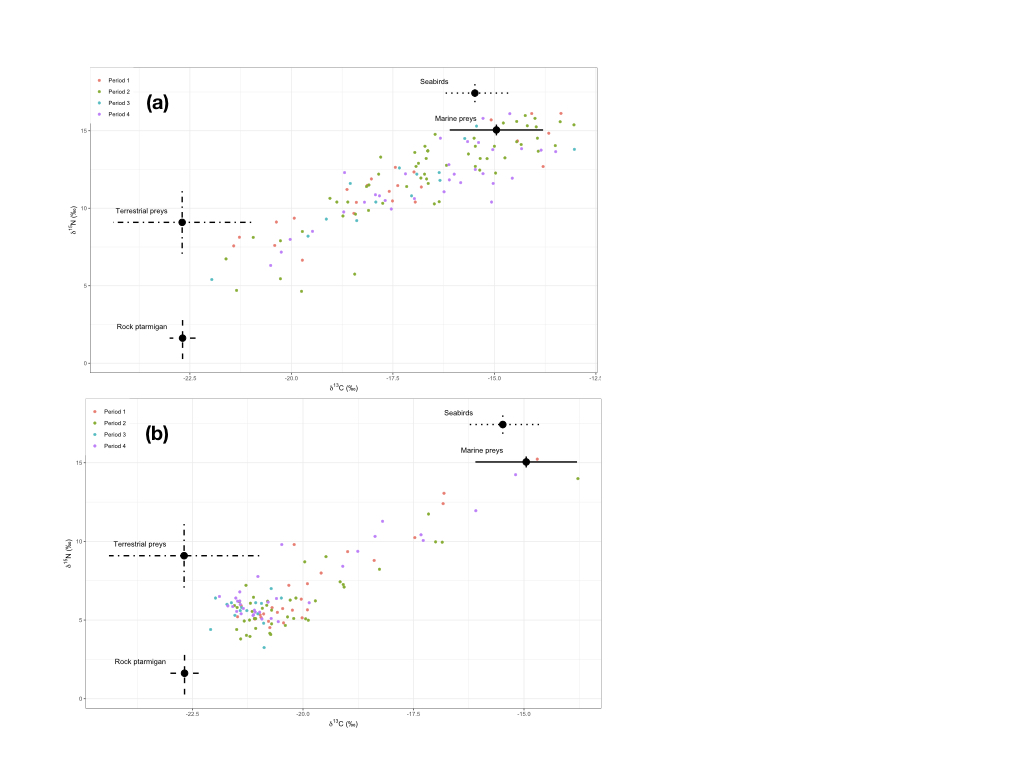

Supplement: S1 Fig — (JPEG) [file pone.0282128.s001.jpeg]

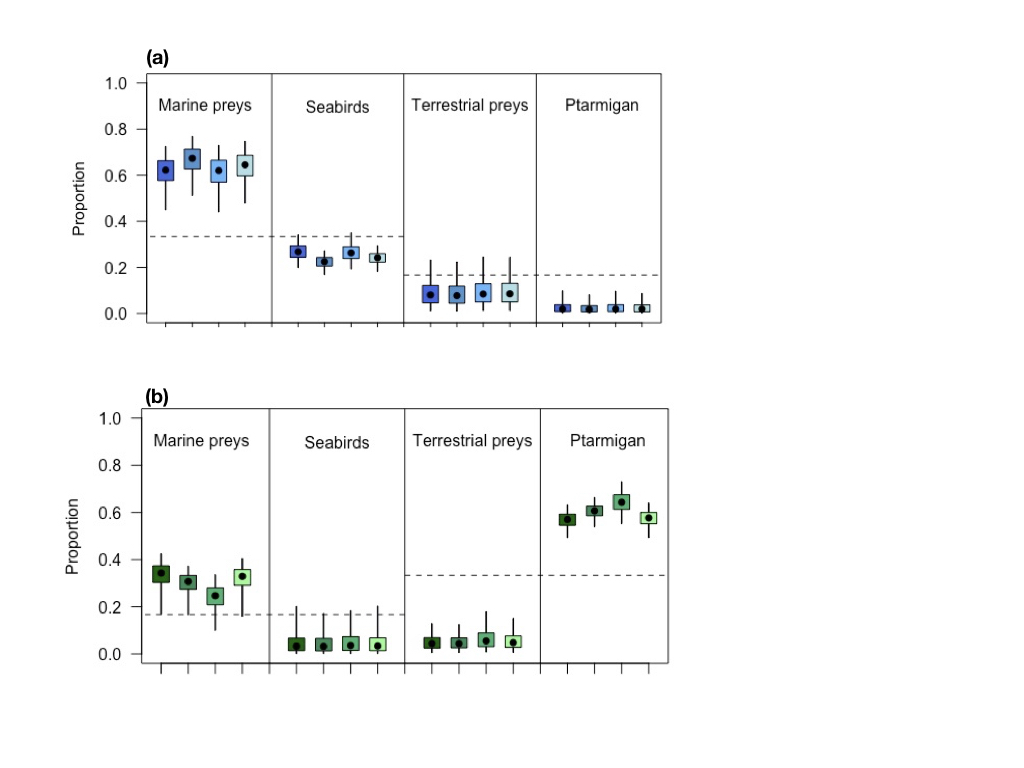

Supplement: S2 Fig — The discrimination factor used for this model was based on a combination of the fractionation values of Arctic fox blood from Lecomte & al. [37], and the blood to collagen variation of wolves from Adams & al. [50]. (JPEG) [file pone.0282128.s002.jpeg]

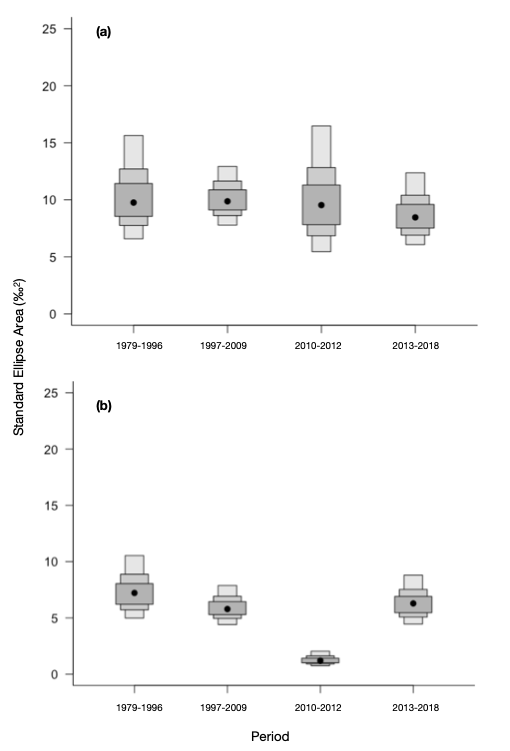

Supplement: S3 Fig — (PNG) [file pone.0282128.s003.png]

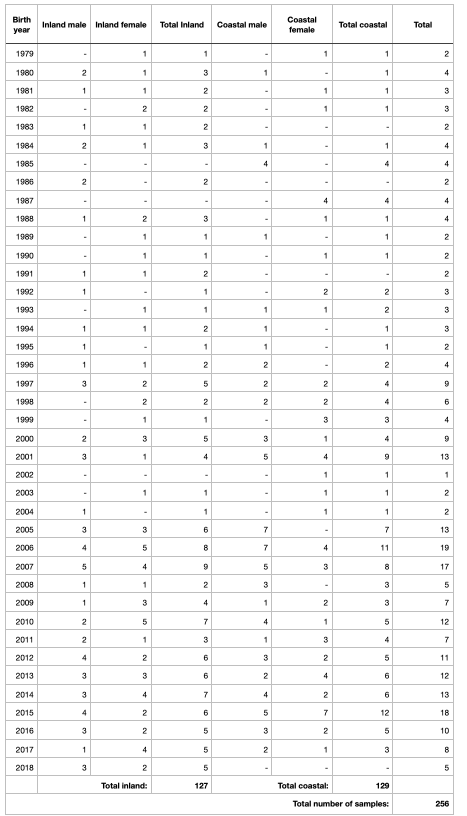

Supplement: S1 Table — (TIF) [file pone.0282128.s004.tif]

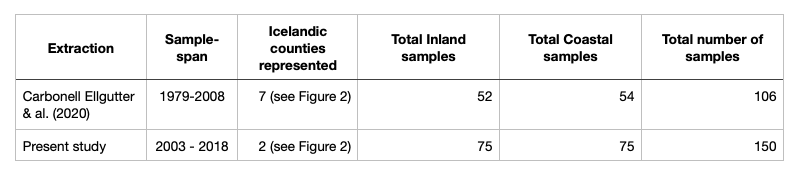

Supplement: S2 Table — Extraction of Carbonell Ellgutter & al. [28] along with the one carried out in the present study. (TIF) [file pone.0282128.s005.tif]
