# Supplementary material for: Long-term responses of Icelandic Arctic foxes to changes in marine and terrestrial ecosystems
Source: PLoS One. 2023 Oct 4;18(10):e0282128. doi: 10.1371/journal.pone.0282128 (PMC10550166; doi:10.1371/journal.pone.0282128)
Supplement: Attachment — Submitted filename: Review to Berthelot et al.pdf [file pone.0282128.s010.pdf]

Review to Berthelot et al.

The manuscript discusses changes in the diet and trophic niches of Arctic foxes over a period of nearly 40 years. The authors examined the stable $\delta^{13}C$ and $\delta^{15}N$ isotope values from the bone collagen of the foxes. The study is very informative and shows very well the changes in diet over the last 40 years and the relationship between foxes and prey availability. However, I have some major and minor comments which should still be considered:

Major comments:

1. Line 127 f: The authors write that the populations were determined using "age cohort analysis and hunting statistics". Could this be a bit more in-depth? What is the relationship between population size and number of kills? Figure 1 gives the impression that the population is always twice the number of kills. I think this is too simplistic. I would ask the authors to elaborate on this.
2. Line 183 ff: The authors write that they extracted collagen according to a specific protocol. It would be desirable to say more about this. Please add a sentence or two describing the steps involved. This will help to classify the values obtained.
3. Line 185: Please enter the laboratory specifications here. This helps to classify how accurate the measurements are. Information about the standards used is also missing.
4. Line 193 ff: This section describes the prey samples. As far as I know, only muscle and egg samples are taken. It would also be important to briefly describe the method of preparation. Can muscle or egg isotopes be compared to collagen isotopes without calibration? If not, the formula for calibration must be mentioned here.
5. Line 214 f: I am not sure with these numbers if this is the correlation of collagen to muscle, or the TEF (Trophic Enrichment Factor) value, or the combination. Please make this clearer. The reference to Fig. 3 is even more confusing as it seems to include TEF values as well. Please clarify what your TEF values are and what the correlation values are.
6. Line 256 ff: Why did the authors use only 300,000 MCMC replicates when the cited manual recommends 1,000,000 MCMC for publications? I also know from many other publications using MixSIAR that 1,000,000 MCMC replicates is the standard size to get a clean running model. In addition, the results of the error diagnostics (Geweke & Gelman-Rubin) to evaluate the usability of the model are missing. These need to be included.
7. Line 269 ff: This section describes how SIBER was used. However, what is missing is the information on how the SEA was calculated. Did the authors use the 40% SEA, which is the core niche, or a 95% SEA, or some other SEA? This information needs to be added. The other niche metrics (e.g., TA and other layman metrics) that can be calculated with SIBER would also be desirable. SIBER also calculates SEAb (Bayesian SEA), which can be used to compare populations and time periods. The different sample sizes do not play a decisive role for this analysis. I strongly recommend including these calculations.

Minor comments:

- Citation in the text: When referring directly to a study, do not say “As [28] showed …” but the name followed by the number. If the authors use Citavi or Endnote, this can be fixed very quickly: Highlight the citation, click Edit, and set it to “display as author(year).
- Line 178: What did the authors mean with “one collagen”?

- Notation of δ^{13}C and δ^{15}N: Please use the international notation for δ^{13}C and δ^{15}N values. The number is superscripted, and the delta is italicized.
- Line 260: Did the authors run the model without priors and is it very different from the model with priors? Maybe the authors can put something like that in the SI.^
- Line 354: The authors should add a small paragraph here about how to deal with missing resources. The selected prey resources cannot reflect 100% of the Arctic fox diet. There may be a resource that was not considered that completely changes the picture. This should be briefly discussed.
- Line 444: This is where the SEAb (or SEAc) comparison mentioned in point 7 would fit in very well to support the statement about the size of the niche.
