# Supplementary material for: Long-term responses of Icelandic Arctic foxes to changes in marine and terrestrial ecosystems
Source: PLoS One. 2023 Oct 4;18(10):e0282128. doi: 10.1371/journal.pone.0282128 (PMC10550166; doi:10.1371/journal.pone.0282128)
Supplement: Attachment — Submitted filename: Answer to reviewers.docx [file pone.0282128.s012.docx]

Dear Editor,

We thank you and the two reviewers for a thorough review of our manuscript. Following the constructive comments of the reviewers, we significantly improved the manuscript. Specifically, we carefully inspected our data according to the quality control criteria recommended by reviewer 2, excluded some values and consequently recalculated all our results. We hope that this new version will be deemed suitable for publication in PLOS One. Below we answer in detail to each comment:

Reviewer 1 :

1) Line 127 f: The authors write that the populations were determined using "age cohort analysis and hunting statistics". Could this be a bit more in-depth? What is the relationship between population size and number of kills? Figure 1 gives the impression that the population is always twice the number of kills. I think this is too simplistic. I would ask the authors to elaborate on this.

*As explained in Unnsteinsdottir et al. (2021) [30], the estimated population size is based on an age cohort model. This model is based on the known harvest data and data from a proportion of the foxes harvested each year that are aged. The model predicts the proportion of each age group that is alive each year, by backtracking age cohorts from live tables, as far back as the oldest aged fox (12 years). The age cohorts are accumulated to get the estimated number of foxes alive each year. This is then corrected for difference in mortality, based on life-table analysis that is done separately for denners (foxes shot at dens) and runners (non-breeding foxes shot away dens). The Icelandic Arctic fox population fits well to this model because hunting is regular, both annually and seasonally. We added two sentences explaining this in the text to figure 1. Regarding the impression from Fig. 1, note that the number of kills and the population size estimated by the model are on different scales.*

2) Line 183 ff: The authors write that they extracted collagen according to a specific protocol. It would be desirable to say more about this. Please add a sentence or two describing the steps involved. This will help to classify the values obtained.

*2) Line 219: more details were added about the protocol.*

3) Line 185: Please enter the laboratory specifications here. This helps to classify how accurate the measurements are. Information about the standards used is also missing.

*3) Line 226: more details were added about the lab’s method and the standards used. For this revised version we also included the measurements from the standards in supplementary table S3.*

4) Line 193 ff: This section describes the prey samples. As far as I know, only muscle and egg samples are taken. It would also be important to briefly describe the method of preparation. Can muscle or egg isotopes be compared to collagen isotopes without calibration? If not, the formula for calibration must be mentioned here.

*4) Line 257: More details were added about the preparation of the muscle and egg samples. Regarding the comparison of different tissues on plots and in the mixing model, we used a diet - consumer discrimination factor (the inference of this factor is described in the section “Data analysis”) to account for the differences between the consumed resource and the collagen of the prey. We did not use any additional calibration or correction, as was the case in numerous earlier papers inferring a species’ diet using stable isotopes (Carbonell Ellgutter & al. 2020, Hall-Aspland & al. 2005, Newsome & al. 2009, Roth 2002).*

5) Line 214 f: I am not sure with these numbers if this is the correlation of collagen to muscle, or the TEF (Trophic Enrichment Factor) value, or the combination. Please make this clearer. The reference to Fig. 3 is even more confusing as it seems to include TEF values as well. Please clarify what your TEF values are and what the correlation values are.

*5) Line 305: The paragraph has been written in a clearer way, and states now the used values for trophic enrichment between arctic fox blood cells and diet, and correction factor between red blood cells and bone collagen estimated in wolves*

6) Line 256 ff: Why did the authors use only 300,000 MCMC replicates when the cited manual recommends 1,000,000 MCMC for publications? I also know from many other publications using MixSIAR that 1,000,000 MCMC replicates is the standard size to get a clean running model. In addition, the results of the error diagnostics (Geweke & Gelman-Rubin) to evaluate the usability of the model are missing. These need to be included.

*6) For this revised version, we rerun all the MixSIAR analyses until convergence or maximum run length. Some runs converged after 1 000 000 replicates (“very long” option in MixSIAR) and for some runs we used the “extreme” option with 3 000 000 replicates. This is now written in the methods (line 397). An assessment of the convergence diagnostics (Geweke & Gelman Rubin diagnostics) is presented in Table S6.*

7) Line 269 ff: This section describes how SIBER was used. However, what is missing is the information on how the SEA was calculated. Did the authors use the 40% SEA, which is the core niche, or a 95% SEA, or some other SEA? This information needs to be added. The other niche metrics (e.g., TA and other layman metrics) that can be calculated with SIBER would also be desirable. SIBER also calculates SEAb (Bayesian SEA), which can be used to compare populations and time periods. The different sample sizes do not play a decisive role for this analysis. I strongly recommend including these calculations.

*7) On the results plot we present the 80% SEA, this is now also mentioned in the figure legend. We also calculated the SEAb to compare the areas, and present these results on Fig S3. This is now mentioned both in the methods (line 425) and in the results (line 533). We chose not to include the other Layman metrics, as they were somewhat redundant.*

8) Citation in the text: When referring directly to a study, do not say “As [28] showed ...” but the name followed by the number. If the authors use Citavi or Endnote, this can be fixed very quickly: Highlight the citation, click Edit, and set it to “display as author(year).

*8) All relevant citations have been modified accordingly.*

9)  Line 178: What did the authors mean with “one collagen”?

*9) Line 213: Spelling mistake: “bone”*

10) Notation of *δ*13C and *δ*15N: Please use the international notation for *δ*13C and *δ*15N values. The number is superscripted, and the delta is italicized.

*10) All the deltas have been set in italic and we checked that superscripts are correctly used.*

11) Line 260: Did the authors run the model without priors and is it very different from the model with priors? Maybe the authors can put something like that in the SI.

*11) For this revised version we repeated all analyses with MixSIAR. We carried out runs for coastal and inland foxes with two different discrimination factors (the one we derived and present in the methods, and the one used by Carbonell-Ellgutter et al. (2020), and using both informative and uniform priors. It is not possible to run a Bayesian analysis without priors, and in the context of a mixing model, all priors are in fact informative. A uniform prior is a prior assuming that all prey are used in equal quantities, whereas we here chose a prior corresponding to previous knowledge of the system with more terrestrial resources for inland foxes and more marine resources for coastal foxes. The results using the different priors were basically identical, showing that they are driven by the data in our case, and not by the choice of the priors. We now mention this in the methods (line 399) and in the results (line 523).*

12) Line 354: The authors should add a small paragraph here about how to deal with missing resources. The selected prey resources cannot reflect 100% of the Arctic fox diet. There may be a resource that was not considered that completely changes the picture. This should be briefly discussed.

*12) Added couple of sentences about that line 641.*

13) Line 444: This is where the SEAb (or SEAc) comparison mentioned in point 7 would fit in very well to support the statement about the size of the niche.

*13) We indeed calculated the SEAb (S3 Fig). This is now described more clearly in the methods and in the results.*

 Reviewer 2:

1)   There is an essential, *non-negotiable* need to include reference to established collagen quality criteria (QC) for bone collagen isotopic compositions. This means reporting and referencing atomic C-to-N ratios (Atomic C:N, hereafter just “C:N”) and the carbon and nitrogen concertation data (%C and %N) that are used calculate them.

a)     C:N ratios have long been a widely accepted standard across disciplines (as per the 3500+ citations of these key QC criteria papers: DeNiro, 1985, Ambrose, 1990) for ensuring that the isotopic compositions (especially for *δ*^13^C) of collagen are not altered by contamination. For collagen from mammalian tissues, Atomic C:N that falls outside the **3.00-3.28** range (see Guiry and Szpak 2020) are contaminated with non-collagenous materials (typically lipids or other proteins) that have been incompletely removed from the bone sample during cleaning and collagen extraction. As these materials have isotopic compositions that can differ drastically (and unpredictably) from that of collagen, samples that fall outside of the acceptable range of Atomic C:N should be discarded, or used with caution and a clear caveat. I should note that, even within the acceptable Atomic C:N range, there can be meaningful impacts of on *δ*^13^C. If you’re worried about this, you can test for it by plotting your *δ*^13^C against Atomic C:N and looking for relationships.

Important notes:

i)      Atomic C:N (as opposed to molecular C:N) is used for collagen C:N work by widely accepted convention and is calculated as follows: (%C/%N)x(14.007/12.011).

ii)    For dataset of the size you are presenting here, these QC data (i.e., C:N, %C, and %N) are typically provided in an ESM in the same table as your isotopic compositions for individual samples (see Comment 2 below).

*We agree that this quality check is important, and that not taking it into account was a weakness of our study. We read Guiry and Szpack (2020) in detail, and as suggested, calculated the atomic C:N ratio for our samples. Plotting them against the δ^13^C values indeed revealed a correlation, although on a figure this appeared mostly drawn by a few outliers. Looking at the distribution of our samples also showed a skewed distribution, with some outliers with high C:N ratios clearly indicating contamination. Removing all samples with a C:N ratio > 3.28 would however correspond to removing 48 samples, nearly 20% of our dataset, making our chronological analysis difficult. Given the C:N ratios calculated from amino-acid compositions in Guiry and Szpack (2020), it appears that theoretically the ratio for mammalian collagen could be up to 3.33. Moreover, applying the approach Guiry and Szpack (2020) used to determine the cut-off for quality check, ie estimating the significance of the correlation between C:N and δ^13^C for subsets of the data and increasing the limit for including data by 0.01 for each analysis, showed a significant relationship already at 3.25. However, in addition to the strength of the correlation, the amount of data is important for whether a test will show significance or not. Thus using such an incremental approach, the power to detect a relationship increases at each step, and partly the significant correlations in our dataset may have been due to its size. Resampling our dataset to 90 samples (ca the number of samples used for significance in Guiry and Szpack (2020), and including samples with C:N up to 3.33 resulted in non-significant correlations in many cases. Thus, we decided to use a cut-off of 3.33 for our data, using the maximum of the ratio calculated based on collagen composition of mammals, and not the threshold determined based on the significance of correlations presented in Guiry and Szpack (2020). We acknowledge in the methods that this may have included samples with  some low level of contamination (lines 238). Given the coefficient of the regression of δ^13^C against C:N was -8.13, a difference in C:N of 0.05 (3.28 to 3.33) would only lead to a change in δ^13^C of 0.4, corresponding to less than 0.2 standard deviations of our δ^13^C values. We excluded 23 samples exceeding this threshold from further analysis.*

2) Results Section. I do not see your raw data included in the manuscript or its supplements. The summary averages presented in your tables cannot stand in place of providing the raw data. Unlike ecology, where in the past original data have sometimes not published, it has become a fundamental requirement for studies using bone collagen data that all isotopic compositions and quality control metrics data are provided so that readers can assess the data for themselves and conduct inter-study comparisons.It is there for an essential requirement that all isotopic data used in this paper is made available to your readership in a supplement.

*2) We added a table with raw δ^13^C and δ^15^N values as well as C:N, %C and %N to the supplementary materials (Table S4).*

3)  Fig 1 caption. Please outline what the blue and green dots show in your caption.

*3) Blue dots represent the number of foxes shot in the coastal population and green dots the number shot in the inland population. We added this information to the figure legend.*

4)  Lines 184-185. More information is need here, particularly with respect to analytical reporting. Your collagen extraction methods derive from the archaeological and paleontological literatures, and this make sense as these are discipline areas with the most experience working out the nuances of preparing and analyzing bone collagen isotopic compositions. However, you have stopped short of what current best practices in this (and other isotope- focused) disciplines require. In that context, I encourage you to consider following current best practices for reporting on bone collagen work as outlined by Szpak et al. (2017). Note that most of this can be done in an electronic supplementary materials section.

a)     Explicitly note how data were calibrated (e.g., a one-, two-, or more-point calibration curve).

b)    With respect to the standards you have used you should note:

i)      what these are,

ii)    which are check standards and which are calibration standards,

iii)  the accepted or long-term average observed isotopic composition for each,

c)     Report tables with the “*n*=”, means (for check standards), and standard deviations (for calibration standards and check standards) for analyses run in each analytical session.

d)    Report tables with the “*n*=”, individual values, means, and standard deviations for all sample replicates.

e)     Consider calculating uncertainty calculation following recommendations from Szpak et al. (2017).

*4) More precision was added about the laboratory specifications (line 225). Additional technical information was added in supplementary Table S3).*

5) Lines 204 to 209. You have glossed over a great deal of complexity here and it would be useful for readers, especially those who may not have an isotope background, to see explicit references to some of the sources of uncertainty built into all these data treatment variables as follows:

a)     How have extraction methods for non-bone collagen tissues been dealt with in terms of potential for lipid contamination? What steps have you taken to ensure the best possible comparability between these data types?

b)    As you mention, you don’t have a direct isotopic offset for fox bone collagen-to-diet and you have come up with a best guess based on stacked inter-tissue offsets. I think perhaps even adding an explicit, brief note that there are uncertainties that can’t be accounted for here is worthwhile, particularly in light of the vary different processes that underly each of the offsets you have come up with.

c)     The Suess correction is not linear. Instead, the rate of change in our atmosphere’s *δ*^13^C is accelerating more and more with each passing year. Because this process continues to build momentum, constant-rate Suess corrections are not well suited for samples from recent decades. I encourage you to consider using a more nuanced approach (Clark et al., 2021) that considers rate of change in your Susse correction. Alternatively, if you can calculate that this rate of change is inconsequential in the context of your interpretations (which, given the wide spread of *δ*^13^C you have observed, is entirely plausible), you could simply add a caveat to that effect.

d)    When and where are the samples used for baseline data from? How have you accounted for potential systematic changes (aside from the Susse effect) in baseline prey isotope compositions? Is it not possible that baseline prey isotopic compositions could vary through time due to changes in prey behavior or environmental conditions (climatic or anthropogenic) influencing relevant C and N cycles? Have you made an attempt to source prey baseline data over the same period of time for which you exampling foxes?

*5) a. All the different types of tissue went through lipid extraction. Therefore we are confident that the determined isotopic composition of the resources are comparable to the isotopic composition of the collagen samples – given the discrimination factor we assume is not too far from the real discrimination.*

*b. A sentence about the uncertainty of the derived discrimination factor was added in the methods (line 311). For the mixing models, we assessed the robustness of our results to the choice of the discrimination factor by repeating the analyses with the factor used by Carbonell Ellgutter et al. (2020).*

*c. The method from Clark & al. 2021 was developed for marine species and may thus not necessarely fit to terrestrial species or species benefiting from both habitats. We did also not find literature in which this method was used for terrestrial species. However, we calculated the Suess effect applying both the method by Clark et al. (2021) and the continuous correction we had used previously. We found very similar results, probably because the time span of our study is still not that large.. Moreover, the continuous correction we applied, that was the same as used by Carbonell Ellgutter & al. (2020), resulted in a very similar amount of annual change as changes measured in tree rings (Belmecheri and Lavergne 2020).*

*d. Prey baseline data has been obtained in more recent years than most of our fox samples (2005-2020 for the preys and 1979-2018 for the foxes). This paper did not account for potential variation in the prey isotopic composition from 1979 to 2005 even though it is possible that prey behavior or environmental conditions influenced their isotopic composition. For all different prey samples, we used a mean value of δ^13^C and δ^15^N, and those are plotted in Fig 3 - corrected for trophic discrimination - with their respective standard deviation to account for uncertainty.*

6)    Line 221. For Figure 3, I suggest the following changes to improve legibility:

a)     Reduce the integer interval for your vertical axis. An interval of, say, 2‰ might make this a bit less cluttered and easier to read.

b)    Add the standard reference materials to relevant axis titles (i.e., AIR, VPDB).

c)     Consider changing the color of one of the fox groups. For instance, if you were to make inland fox “x” symbols black, this would help them ‘pop’ a bit better. As it stands, in some areas of the plot, the data forms a cloud that is difficult to make sense of.

*6) Fig 3 has been modified accordingly.*

7)    Lines 243-251. Somewhere (elsewhere would be fine if you prefer) you should deal with the potential for freshwater food use by: 1) acknowledging that freshwater foods represent a tremendous range of potential variation in source isotope values (for review see Guiry, 2019) and, 2) outline the extent to which such resources might have been available to foxes. I suspect, based on the fact that you haven’t touched on this as a possibility, that there is a low likelihood that foxes have had substantial access to freshwater resources. However, reference to the fact your aware of this as a potential interpretive issue would be useful for your readers who are not familiar with there are (and could therefore misunderstand this as an omission of potentially relevant baseline source data).

*7) We agree that freshwater food would have added to the range of potential variation in source isotopic values. However, in Iceland, despite several diet studies carried out with arctic foxes, use of freshwater resources has not been reported. Moreover, as bone collagen has a very low turnover and mostly represents the average diet in the first year, we think it is unlikely that the rare consumption of a freshwater organism would change the isotopic composition red fox bone collagen majorly. However, to not omit this completely, we mention now freshwater food in the discussion (line 643)*

8)    Line 310. “that” should be “than”. Note that, though I have seen several potential word choice issues, I have not been made a systematic attempt to comment on these. I suggest you go over the manuscript in detail prior to resubmission.

*8) We went through the manuscript in detail and tried to fix all the small mistakes.*

9)    Line 346. Here and throughout the manuscript (e.g., line 379, 458) please be more specific about what kind of values you are referring to. It is not good practice to use vague terminologies like “nitrogen ratios”, “carbon ratios”, or “carbon signature”. In the case of “nitrogen ratios”, for instance, this is because this could technically refer to a ratio involving the concentration of nitrogen (i.e., %N, which should also be reported as a key QC metrice (see Comment 1 above) or nitrogen isotope compositions (i.e., *δ*^15^N). I understand what you mean here but it is important to use correct terminology to help avoid confusion among readers with less experience in this area (for a review on terminology see Coplen, 2011).

*9) We clarified the terminology used throughout the manuscript.*

Cited literature:

Belmecheri S, Lavergne A. Compiled records of atmospheric CO2 concentrations and stable carbon isotopes to reconstruct climate and derive plant ecophysiological indices from tree rings. Dendrochronologia. Volume 63. 2020. 125748. ISSN 1125-7865,

Carbonell Ellgutter JA, Ehrich D, Killengreen ST, Ims RA, Unnsteinsdóttir ER. Dietary variation in Icelandic arctic fox (*Vulpes lagopus*) over a period of 30 years assessed through stable isotopes. Oecologia. 2020. 192, 403–414.

Hall-Aspland, S. A., Rogers, T. L., & Canfield, R. B. (2005). Stable carbon and nitrogen isotope analysis reveals seasonal variation in the diet of leopard seals. *Marine Ecology Progress Series*, *305*, 249–259.

Newsome, S. D., Tinker, M. Tim, Monson, D. H., Oftedal, O. T., Ralls, K., Staedler, M. M., Fogel, M. L., & Estes, J. A. (2009). Using stable isotopes to investigate individual diet specialization in California sea otters ( Enhydra lutris nereis ). *Ecology*, *90*(4), 961–974.

Roth, J. D. (2002). Temporal variability in arctic fox diet as reflected in stable-carbon isotopes; the importance of sea ice. *Oecologia*, *133*(1), 70–77.

Unnsteinsdóttir ER. Refastofninn réttir úr sér, Fréttatilkynning frá Náttúrufræðistofnun Íslands. 2021. Icelandic.
