# Supplementary material for: Long-term responses of Icelandic Arctic foxes to changes in marine and terrestrial ecosystems
Source: PLoS One. 2023 Oct 4;18(10):e0282128. doi: 10.1371/journal.pone.0282128 (PMC10550166; doi:10.1371/journal.pone.0282128)
Supplement: Attachment — Submitted filename: Answer to reviewers2.docx [file pone.0282128.s013.docx]

Dear Editor,

We thank you and the two reviewers for the feedbacks on our reviewed manuscript. We were glad our modifications satisfied you almost entirely. We modified the manuscript in agreement with your latest comments, and we hope that this new version will be deemed suitable for publication in PLOS One.

Below we answer in detail to each comment:

NB: The lines correspond to the line number on the document with track change.

Reviewer 1:

1-3) The lines the authors specified do not match either of the two uploaded versions.

*1-3) We apologize for the inconvenience. We noticed that this issue made our information about missing resources difficult to retrieve. As pointed out by the editor, the missing resources were dealt with line 427 of the previous version of our manuscript without track change (see now line 438 on the document with track change). To make it even clearer, we added more precisions about the consequences of those missing resources.*

2) The authors wrote that they have more information about Geweke and Gelman Rubin diagnostics in table S6. But this information is very incomplete. It would be better to give the specifications. How many variables have been tested? How many of them were in the Gelman Rubin diagnostic above 1.05? How many variables were unequal in the Geweke disgnostic and in which chains (at best less than 5%)? This information is important to measure the quality of Bayesian models using MCMC and must be published.

*2) The results of the diagnostics (Geweke and Gelman Rubin) have been added to table S6.*

Academic editor:

- Please answer comment 2) of reviewer 1 and add the results of the statistical tests in table S6.

*The results of the diagnostics (Geweke and Gelman Rubin) have been added to table S6.*

- Line 201-202: Are all the standards quoted equivalent to international reference material such as USGS or is there any internal (=inhouse) reference material?

*All the standards quoted are equivalent to international reference material. This is now specified in the text as well (line 201) .*

- Line 208-209 “We thus included 25 samples with a C:N ratio between 2.28 and 3.33” while the authors convincingly argue about considering isotopic results on collagen with C:N atomic ratio up to 3.33, I do not see any argument to keep samples with C:N below 3.0 as recommended by Guiry &Szpak (not Szpack as in table S4) 2020 and could not spot any atomic C:N under 3.09 in your table S4 anyway. I guess you confused with the molecular C/N ratio of column J. Please specify that J column is a molecular C/N ratio and K column is an atomic C/N ratio (named C:N by convention), avoiding further confusion, and amend your main text accordingly.

*We apologize for the confusion and attribute it to a mistake: we meant samples with an atomic C:N ration between 3.28 and 3.33, as our point was to justify the use of samples with atomic C:N > 3.28 (which is the high limit of the range advised by Guiry & Szpak 2020). We corrected this in table S4 as well as the text (see line 207-211).*

- Line 385-386: “no statistical evidence for changes in nitrogen isotopic composition, and the changes in δ13C” please replace by “ no statistical evidence for changes in δC and δN values” for sake of consistency

*Line 392: The sentence has been modified as suggested.*

- Line 429-431: I guess it is where you consider missing resources as recommended by reviewer 1. Could you add a sentence about the consequences of missing terrestrial resources in your reconstruction, as you did for freshwater resources, to better address the point of the reviewer? In other words, beside t the pink-footed goose, which other terrestrial species contribution may have been over-or underestimated?

*Line 438: More details have been added regarding the lack of some marine and terrestrial sources and their potential consequences.*
