# Supplementary material for: Long-term responses of Icelandic Arctic foxes to changes in marine and terrestrial ecosystems
Source: PLoS One. 2023 Oct 4;18(10):e0282128. doi: 10.1371/journal.pone.0282128 (PMC10550166; doi:10.1371/journal.pone.0282128)
Supplement: Attachment — Submitted filename: Reviewer Comments - Guiry March 2 2023.docx [file pone.0282128.s011.docx]

**Title Reviewed:** *Long-term responses of Icelandic Arctic foxes to changes in marine and terrestrial ecosystems*

**Recommendation:** Major Revisions

Dear Dr. Drucker,

Thank you kindly for the invitation to review “Long-term responses of Icelandic Arctic foxes to changes in marine and terrestrial ecosystems” for *PLOS1* (manuscript number: PONE-D-23-03420). This paper explores isotopic compositions of collagen extracted from Arctic fox bones of known date and origin in Iceland to reconstruct dietary shifts over a period almost 70 years beginning in 1950. Data are interpreted in the context of baseline prey and long-term biological and ecological observations of fox food web taxa to reveal diachronic differences in the foraging ecology of foxes with different habitat preferences.

The overall approach employed by the authors appears sound (though I leave evaluation of their mixing model use to other reviewers) and I believe the results and interpretations could be of value for future researchers in a wide range of disciplines, topical areas, and time periods, well outside this study’s spatiotemporal scope. For instance, beyond the recent historical ecology focus of this study, by incorporating ancient samples (that can be abundant at Arctic archaeological sites), research on the stable isotope compositions of fox bone collagen has potential to contribute to broader narratives about a wide range of human-environmental relationships (not just climate change) through the Holocene. The impressive sample presented here joins a growing fox-centered literature and could help to provide a valuable springboard for expanding this kind of work. I am therefore delighted to indicate that this paper will likely be publishable, pending close and detailed consideration of the comments I have offered below.

Having offered an overall positive assessment of the study I need to highlight some critical issues that, in my opinion, render the paper unpublishable until they are fully resolved. While I consider all of the comments offer below relevant, I am referring in particular to Comments 1 and 2, which deal with the fact that the paper provides none of its raw data and give no quality control (QC) metrics. Omitting data in this way is no longer acceptable (even for ecologists) and I can see no reason (especially given the option of inclusion as an ESM table) that these data should be made full available. As it stands, while I’m excited about the paper’s approach and interpretations, I am unable to evaluate the validly of their core data and this has prevented me from fully evaluating it.

Given the serious situation that omission of fundamental data this this represents, my recommendation is ‘major revision’, though I recognize that the comments offered below could be satisfied with relatively little change to the manuscript’s text. If you have any questions about these comments, please do not hesitate to contact me.

Sincerely,

Dr. Eric Guiry, School of Archaeology and Ancient History, University of Leicester

**Reviewer Comments**

This manuscript was a pleasure to read and I would like to commend the authors on what appears to be an exciting study. That said, I recommend the following changes, which are aimed at enhancing the paper’s ecological and isotopic background and clarifying language around some interpretations. Note that I consider compliance with Comment's 1 and 2 to be critical to the paper's publishability.

**General Comments**

1) Methods Section. There is an essential, *non-negotiable* need to include reference to established collagen quality criteria (QC) for bone collagen isotopic compositions. This means reporting and referencing atomic C-to-N ratios (Atomic C:N, hereafter just “C:N”) and the carbon and nitrogen concertation data (%C and %N) that are used calculate them. I cannot stress the importance of ensuring that these data are included for all samples enough. This is the primary means by which the data quality all bone collagen samples (modern or archaeological) is evaluated, a convention that has been widely accepted by the those working with bone collagen stable isotope research for decades. In that context, not only is it key that you include these QC data to follow rudimentary standards of data hygiene practices, but including these QC data will also likely be key for ensuring more interdisciplinary engagement with your study. For instance, many broader meta-analyses (of the kind that are increasingly making big discoveries in retrospective animal isotopic compositions studies today) would exclude data that has no associated QC information. Below, I have offered a brief overview of C:N ratios with reference to the some of the nuances of which approach is best suited for work with modern samples.
   a) C:N ratios have long been a widely accepted standard across disciplines (as per the 3500+ citations of these key QC criteria papers: DeNiro, 1985, Ambrose, 1990) for ensuring that the isotopic compositions (especially for *δ*^13^C) of collagen are not altered by contamination. While these QC criteria were developed initially for ancient samples, they have long been used in contemporary ecology and have recently been revised (Guiry and Szpak, 2020) to further improve their ability to ensure data from modern samples are more accurate. In a nutshell, unlike, more commonly analyzed materials from modern specimens, such as muscle, collagen has a highly conserved, and specific Atomic C:N across taxa, which can provide a robust marker for contamination detection. For collagen from mammalian tissues, Atomic C:N that falls outside the **3.00-3.28** range (see Guiry and Szpak 2020) are contaminated with non-collagenous materials (typically lipids or other proteins) that have been incompletely removed from the bone sample during cleaning and collagen extraction. As these materials have isotopic compositions that can differ drastically (and unpredictably) from that of collagen, samples that fall outside of the acceptable range of Atomic C:N should be discarded, or used with caution and a clear caveat. I should note that, even within the acceptable Atomic C:N range, there can be meaningful impacts of on *δ*^13^C. If you’re worried about this, you can test for it by plotting your *δ*^13^C against Atomic C:N and looking for relationships. Important notes:
      i) Atomic C:N (as opposed to molecular C:N) is used for collagen C:N work by widely accepted convention and is calculated as follows: (%C/%N)x(14.007/12.011).
      ii) For dataset of the size you are presenting here, these QC data (i.e., C:N, %C, and %N) are typically provided in an ESM in the same table as your isotopic compositions for individual samples (see Comment 2 below).
2) Results Section. I do not see your raw data included in the manuscript or its supplements. The summary averages presented in your tables cannot stand in place of providing the raw data. Unlike ecology, where in the past original data have sometimes not published, it has become a fundamental requirement for studies using bone collagen data that all isotopic compositions and quality control metrics data are provided so that readers can assess the data for themselves and conduct inter-study comparisons. Globally, in the past 35 years, hundreds of thousands of isotopic compositions from extracted, purified bone collagen have been published in full in the archaeological, historical ecological, and paleontological literatures and data from previous studies are routinely used for comparative purposes to support interpretations in new studies. It is critical that we make our data as freely available and useful to future researchers as possible. A key reason for this is that isotopic analyses are a destructive process – the samples we cut from bones and then analyse can never be analyzed again. It therefore behoves us all to ensure that our data can contribute to wider discussions in the future. If you do not provide *δ*^13^C and *δ*^15^N values from extracted bone collagen, future studies will not be able to link datasets with yours – this may serve to discourage inclusion of your work in future debates. In other words, because isotopic analyses destroy unique, un-replaceable samples we have an ethical responsibility to make all raw data freely available. It is there for an essential requirement that all isotopic data used in this paper is made available to your readership in a supplement.

Specific Comments

3) Fig 1 caption. Please outline what the blue and green dots show in your caption.
4) Lines 184-185. More information is need here, particularly with respect to analytical reporting. Your collagen extraction methods derive from the archaeological and paleontological literatures, and this make sense as these are discipline areas with the most experience working out the nuances of preparing and analysing bone collagen isotopic compositions. However, you have stopped short of what current best practices in this (and other isotope- focused) disciplines require. In that context, I encourage you to consider following current best practices for reporting on bone collagen work as outlined by Szpak et al. (2017). Note that most of this can be done in an electronic supplementary materials section.
   a) Explicitly note how data were calibrated (e.g., a one-, two-, or more-point calibration curve).
   b) With respect to the standards you have used you should note:
      i) what these are,
      ii) which are check standards and which are calibration standards,
      iii) the accepted or long-term average observed isotopic composition for each,
   c) Report tables with the “*n*=”, means (for check standards), and standard deviations (for calibration standards and check standards) for analyses run in each analytical session.
   d) Report tables with the “*n*=”, individual values, means, and standard deviations for all sample replicates.
   e) Consider calculating uncertainty calculation following recommendations from Szpak et al. (2017).
5) Lines 204 to 209. You have glossed over a great deal of complexity here and it would be useful for readers, especially those who may not have an isotope background, to see explicit references to some of the sources of uncertainty built into all these data treatment variables as follows:
   a) How have extraction methods for non-bone collagen tissues been dealt with in terms of potential for lipid contamination? What steps have you taken to ensure the best possible comparability between these data types?
   b) As you mention, you don’t have a direct isotopic offset for fox bone collagen-to-diet and you have come up with a best guess based on stacked inter-tissue offsets. I think perhaps even adding an explicit, brief note that there are uncertainties that can’t be accounted for here is worthwhile, particularly in light of the vary different processes that underly each of the offsets you have come up with. For instance, while there are large isotopic offsets between both the *δ*^13^C and *δ*^15^N of an animal’s collagen and its diet, the underlying processes behind these and the extent to which they have been studied differs in some big ways. Diet-consumer bone collagen shifts in *δ*^15^N result from trophic enrichment of ^15^N and this has undergone a lot of study. The offset value you have selected here is broadly in line with results from relevant meta-analyses. By contrast, diet-consumer bone collagen shifts in *δ*^13^C are not the result of large trophic enrichment shifts, but instead derive largely from intra-individual, inter-tissue differences that are inherently difficult to study (due to the slow turnover of bone collagen and length of time needed for controlled feeding studies). In that context, it is more challenging to see if the offset you have come up with for *δ*^13^C is in the right ballpark.
   c) The Susse correction is not linear. Instead, the rate of change in our atmosphere’s *δ*^13^C is accelerating more and more with each passing year. Because this process continues to build momentum, constant-rate Suess corrections are not well suited for samples from recent decades. I encourage you to consider using a more nuanced approach (Clark et al., 2021) that considers rate of change in your Susse correction. Alternatively, if you can calculate that this rate of change is inconsequential in the context of your interpretations (which, given the wide spread of *δ*^13^C you have observed, is entirely plausible), you could simply add a caveat to that effect.
   d) When and where are the samples used for baseline data from? How have you accounted for potential systematic changes (aside from the Susse effect) in baseline prey isotope compositions? Is it not possible that baseline prey isotopic compositions could vary through time due to changes in prey behavior or environmental conditions (climatic or anthropogenic) influencing relevant C and N cycles? Have you made an attempt to source prey baseline data over the same period of time for which you exampling foxes?
6) Line 221. For Figure 3, I suggest the following changes to improve legibility:
   a) Reduce the integer interval for your vertical axis. An interval of, say, 2‰ might make this a bit less cluttered and easier to read.
   b) Add the standard reference materials to relevant axis titles (i.e., AIR, VPDB).
   c) Consider changing the color of one of the fox groups. For instance, if you were to make inland fox “x” symbols black, this would help them ‘pop’ a bit better. As it stands, in some areas of the plot, the data forms a cloud that is difficult to make sense of.
7) Lines 243-251. Somewhere (elsewhere would be fine if you prefer) you should deal with the potential for freshwater food use by: 1) acknowledging that freshwater foods represent a tremendous range of potential variation in source isotope values (for review see Guiry, 2019) and, 2) outline the extent to which such resources might have been available to foxes. I suspect, based on the fact that you haven’t touched on this as a possibility, that there is a low likelihood that foxes have had substantial access to freshwater resources. However, reference to the fact your aware of this as a potential interpretive issue would be useful for your readers who are not familiar with there are (and could therefore misunderstand this as an omission of potentially relevant baseline source data).
8) Line 310. “that” should be “than”. Note that, though I have seen several potential word choice issues, I have not been made a systematic attempt to comment on these. I suggest you go over the manuscript in detail prior to resubmission.
9) Line 346. Here and throughout the manuscript (e.g., line 379, 458) please be more specific about what kind of values you are referring to. It is not good practice to use vague terminologies like “nitrogen ratios”, “carbon ratios”, or “carbon signature”. In the case of “nitrogen ratios”, for instance, this is because this could technically refer to a ratio involving the concentration of nitrogen (i.e., %N, which should also be reported as a key QC metrice (see Comment 1 above) or nitrogen isotope compositions (i.e., *δ*^15^N). I understand what you mean here but it is important to use correct terminology to help avoid confusion among readers with less experience in this area (for a review on terminology see Coplen, 2011).

**References**

Ambrose, S.H. 1990. Preparation and characterization of bone and tooth collagen for isotopic analysis. *Journal of Archaeological Science* 17: 431-451.

Clark, C.T., Cape, M.R., Shapley, M.D., Mueter, F.J., Finney, B.P. & Misarti, N. 2021. SuessR: Regional corrections for the effects of anthropogenic CO2 on δ13C data from marine organisms. *Methods in Ecology and Evolution* 12: 1508-1520.

Coplen, T.B. 2011. Guidelines and recommended terms for expression of stable‐isotope‐ratio and gas‐ratio measurement results. *Rapid communications in mass spectrometry* 25: 2538-2560.

DeNiro, M.J. 1985. Postmortem preservation and alteration of in vivo bone collagen isotope ratios in relation to palaeodietary reconstruction. *Nature* 317: 806-809.

Guiry, E. 2019. Complexities of stable carbon and nitrogen isotope biogeochemistry in ancient freshwater ecosystems: implications for the study of past subsistence and environmental change. *Frontiers in Ecology and Evolution* 7.

Guiry, E. & Szpak, P. 2020. Quality Control for Modern Bone Collagen Stable Carbon and Nitrogen Isotope Measurements. *Methods in Ecology and Evolution*.

Szpak, P., Metcalfe, J.Z. & Macdonald, R.A. 2017. Best practices for calibrating and reporting stable isotope measurements in archaeology. *Journal of Archaeological Science: Reports* 13: 609-616.
